# Reproductive plasticity and environmental tolerance of invasive African catfish (*Clarias gariepinus*) in a tropical Brazilian river

**DOI:** 10.1111/jfb.70389

**Published:** 2026-03-19

**Authors:** Michelle Torres Dumith, Alejandra Filippo Gonzalez Neves dos Santos

**Affiliations:** ^1^ Applied Ecology Laboratory (LEA) and Ichthyology Experimental Animal Facility, Department of Animal Science and Sustainable Social‐Environmental Development, Faculty of Veterinary Medicine Fluminense Federal University Niterói Brazil

**Keywords:** bioinvasion, *Clarias gariepinus*, environmental conditions, reproduction, reproductive indices

## Abstract

Invasive alien species (IAS) pose a significant threat to biodiversity, particularly when endowed with high ecological and reproductive plasticity. The African catfish, *Clarias gariepinus*, is one such species, widely recognized for its ability to colonize and establish in diverse tropical ecosystems. This study evaluated the reproductive success of *C. gariepinus* in a tropical environment by examining gonadal maturation stages, reproductive indices and water physicochemical variables associated with reproduction. The analysis revealed a population predominantly composed of adult individuals at various reproductive stages, with a higher concentration upstream during the dry season. The species exhibited continuous reproduction throughout the year, irrespective of body size, demonstrating both high fecundity and notable environmental flexibility. Dobriyal's Reproductivity Index (DI) proved effective in identifying gonadal maturation patterns independently of somatic size and is particularly useful under conditions of limited sampling. Statistical analyses revealed a strong association between DI and key physicochemical parameters (i.e., particularly elevated dissolved oxygen concentrations and pH values within optimal ranges) which collectively influence species distribution and reproductive success. These findings underscore reproductive plasticity and environmental tolerance as pivotal strategies underpinning the invasive potential of African catfish in tropical systems. We conclude that ongoing monitoring and targeted management interventions are crucial for mitigating the ecological impacts of *C. gariepinus* and alleviating pressure on native fish communities.

## 1 INTRODUCTION

Invasive alien species (IAS) can proliferate unchecked, causing significant negative impacts on the ecosystem (Santos et al., 2018). These invaders can directly compete with native species for resources, feeding on them and contributing to the impoverishment and homogenization of ecosystems (Clavero & García‐Berthou, 2006). This represents one of the greatest threats to the reduction in and degradation of habitats, negatively impacting biodiversity and ecosystem processes (Colin et al., 2018; Erarto & Getahun, 2020; Gallardo et al., 2019; Katsanevakis et al., 2014). In aquatic environments, the presence of IAS is associated with human activities (Gallardo et al., 2015; Havel et al., 2015), with aquaculture being an important vector for the introduction and spread of these species (Gozlan et al., 2010; Lee et al., 2017). Effective control and management of these invaders are essential to preserve the integrity of aquatic ecosystems and minimize adverse effects on native fauna and flora (Carey et al., 2012; Woodford et al., 2016).

*Clarias gariepinus* (Burchel, 1822) (Siluriformes, Clariidae) is one of the most globally cultivated species, being the primary catfish species produced and consumed in Africa and Asia, and some parts of Europe (Dauda et al., 2018). With tasty, boneless flesh and low ash content (Adebayo et al., 2016; Karim et al., 2017; Oladipo & Bankole, 2013), these nutritional characteristics are important for consumption in the aquaculture market (Papuc et al., 2019). From a commercial perspective, African catfish exhibit rapid growth that can tolerate low concentrations of dissolved oxygen in the water (Belão et al., 2011; Moussa, 1956), and can be stocked at high densities in aquaculture (Kucharczyk et al., 2019). However, in captivity, it loses its natural reproductive ability (Romanova et al., 2020; Romanova, Lyubomirova, Romanov, Mukhitova, Shlenkina, Shadieva, & Galushko, 2018), requiring hormonal induction techniques to supply larvae and juveniles for the industry (Addo et al., 2022; FAO, 2022). Like other species used in intensive aquaculture (such as tilapia and carp), *C. gariepinus* exhibits high levels of hardiness, dietary and ecological plasticity that facilitate its escape and establishment in natural environments (Dumith & Santos, 2023; Vitule et al., 2009). Even so, the environmental and physicochemical characteristics of the water remain crucial for the reproductive expression of the species, both in cultivation and in natural environments (Ariole et al., 2021; Legendre et al., 1996; Santi et al., 2017).

These hardiness attributes (environmental tolerance, aggressive behaviour, a generalist diet and high fecundity) render the African catfish a species with high invasive potential (Cambray, 2003; Jarić et al., 2015; Vitule et al., 2009). Its accidental or deliberate introduction has led to the establishment of naturalized populations in Asia and Central and South America. For example, in the Malay Peninsula, the invasion has caused negative impacts on its congener *Clarias batrachus*, due to synergistic competition between habitats and trophic factors (Low et al., 2022). Another example associated with the invasion of the African catfish is related to the negative impact on the macroinvertebrate community, resulting in a decrease in the diversity, richness and biomass of vulnerable taxa in South Africa (Kadye & Booth, 2012). Thus, the species is characterized as a prolific predator, competing for food and altering the structure of the food chain, being considered a serious threat to native fish fauna as a source of negative effects on aquatic ecosystems (Radhakrishnan et al., 2011). In Latin America and Brazil, the species overcame invasion barriers by escaping from aquaculture facilities or through illegal introduction (Weyl et al., 2016), established since 1986 (Nóbrega et al., 2024). In Brazil, studies on competitive or predatory impacts in invaded ecosystems are scarce (Weyl et al., 2016); however, current Brazilian legislation prohibits its introduction into certain river basins (Vitule et al., 2006, 2009; Weyl et al., 2016).

Parameters such as dissolved oxygen, pH and temperature are recognized as critical regulators of fish reproduction, functioning as environmental filters or facilitators of invasion (Bhatnagar & Davi, 2013; Heath, 2018). However, despite the widespread distribution of *C. gariepinus* in invaded habitats, the ecological mechanisms underpinning their reproductive success in degraded environments remain poorly explored. Moreover, established populations of African catfish in different tropical regions have exhibited high plasticity even under polluted conditions (Weyl et al., 2016). The species' occurrence in systems subject to pollution, deforestation, hydrological alterations and other anthropogenic pressures raises questions regarding its capacity to reproduce under suboptimal environmental conditions (Fistarol et al., 2015; Low et al., 2022; Sreekanth et al., 2022). In this context, the present study offers an opportunity to investigate which reproductive traits may facilitate invasion in tropical environments and thereby disrupt the structure of native aquatic communities.

The present study aims to investigate the reproductive biology of a naturalized African catfish population in a tropical estuarine environment, with a focus on (1) the distribution of gonadal maturation stages, (2) key reproductive indices and (3) their relationships with water physicochemical variables, thereby identifying environmental conditions that promote species reproductivity outside of controlled systems. Furthermore, this work seeks to elucidate the biological patterns underpinning the success of reproductively robust invaders and to provide scientific support for monitoring and management strategies in vulnerable aquatic ecosystems.

## 2 METHODS

### 2.1 Study area and sample design

**FIGURE 1:**
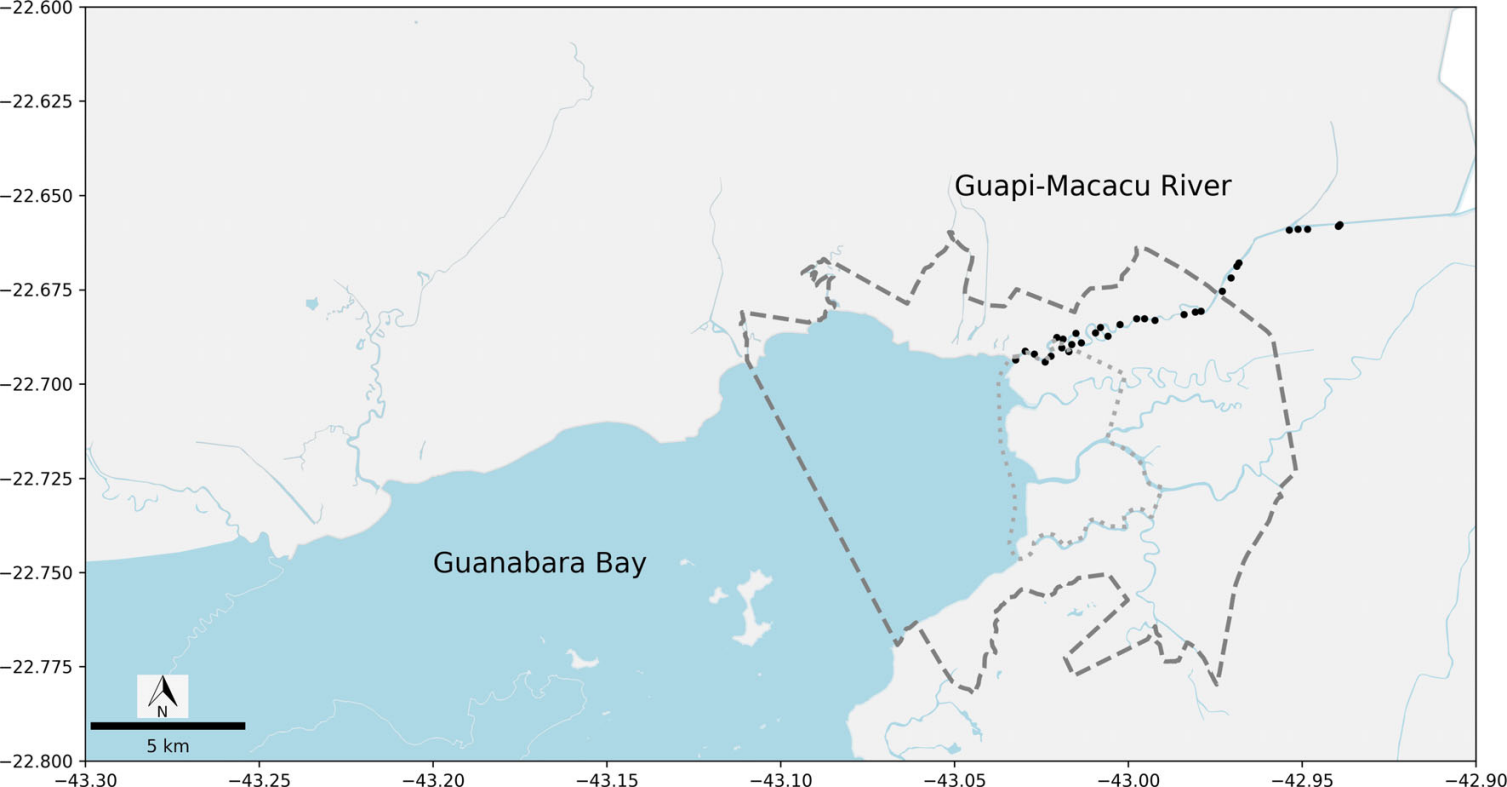
Sampling area map (collection points) in the Guapi‐Macacu River (black points along the river), with the delineation of the Guapimirim Environmental Protection Area (dashed line) in Rio de Janeiro, Brazil.

The sampling area was in the Guapimirim Environmental Protection Area (EPA), located in southeastern Brazil (Figure 1), in a region adjacent to Guanabara Bay. In this area, local accounts indicate that feral *C. gariepinus* originated from a fish farm that has been inactive since the early 1990s, suggesting that the population has been naturalized for more than 20 years. In addition, local artisanal fishers report that the occurrence of African catfish has become more frequent than that of native fish species, which were previously more common. However, proximity to industrial complexes, insufficient sanitary sewage treatment and hydrological exchange with Guanabara Bay have resulted in numerous water‐quality issues (Roberto Da Costa et al., n.d.; Fistarol et al., 2015; Vicente et al., 2016), thereby exacerbating the region's environmental degradation (Fistarol et al., 2015; Monteiro et al., 2012). The area is characterized by extensive mangrove vegetation interwoven with a network of rivers and channels subject to seasonal inundation (Fries et al., 2019). These hydrological dynamics confer high connectivity among aquatic habitats and generate a complex spatial structure (de Ferreira Barreto et al., 2019), which both facilitates dispersal and complicates fish capture.

Sampling was concentrated in the Guapi‐Macacu River, which exhibits the highest discharge and greatest length among the rivers within the study area (de Ferreira Barreto et al., 2019; Fries et al., 2019). Two field sampling campaigns were conducted, covering the most distinct seasonal periods in the region (Henrique et al., 2009; Silva et al., 2009, 2018) February–March, corresponding to the rainy season, and August–September, corresponding to the dry season, in 2018. These periods were selected because the African catfish exhibits different reproductive periods that vary based on its geographic location (Goos & Kichter, 1996). However, the literature also suggests that the species is more closely associated with photoperiod, temperature and water flow, and may present multiple spawning cycles throughout the year (Bruton, 1979; CLAY, Clay, 1979). Conversely De Graaf and Janssen (1996) observed that the species show maturation processes during both the rainy season (October to May) and the dry season (June to September) in unprotected culture ponds, following seasonal fluctuations in rainfall and air temperature, which coincides with the seasonal periods considered in this study.

Sampling was conducted at 32 sampling stations, comprising a total of 10 downstream sites, 12 sites in the intermediate area and 10 upstream sites. A variety of fishing gear was employed, including gill nets (60 × 1.5 m) with different mesh‐sizes (15, 30 and 45 mm between adjacent knots), cast nets, pots and traps (40 cm height × 35 cm diameter nylon traps), hooks and lines. At each sampling site, fishing gear was used simultaneously by different operators, with three replicates (each lasting 1 h) at each site, both during daylight and at night, totalling 64 sampling events (Figure 1). Nets and trap gear were deployed at the sampling stations for at least 12 h, depending on the river stretch, being installed at night and retrieved during the daytime. To encompass the physiographic diversity of the Guapi‐Macacu River from its mouth to upstream reaches, we measured seven abiotic variables to identify key water‐quality parameters hypothesized to influence the exotic reproduction species. Percentages of vegetation cover and the most prominent physiographic characteristics along the river stretches were also recorded by the observer at each sampling point (Rios et al., 2006; Smith et al., 2005). A YSI Multiparameter Sonde (Model 6600) was used in situ alongside each fishing gear deployment to record water temperature (°C), pH, turbidity (NTU), dissolved oxygen (mg/L), salinity (PSU), phycocyanin (RFU) and chlorophyll (µg/L). Immediately following capture, specimens were labelled, chilled on ice in the field and transported to the Applied Ecology Laboratory at Federal Fluminense University for processing.

### 2.2 Laboratory procedures

In the laboratory, using the relevant taxonomic references (Bénech et al., 1992; Figueiredo & Menezes, 2015; Froese & Pauly, 2021; Roberts, 1989), 30 specimens of *C. gariepinus* were identified, weighed on a precision balance (0.001 g) and measured for total length (TL) and standard length (SL) to the nearest millimetre. Subsequently, each specimen was eviscerated to remove the liver and gonads, which were then weighed and assessed for sex and macroscopic gonadal maturation stage. Sex determination was performed by observation under a stereomicroscope based on the macroscopic morphology of the gonads (Souza & Chaves, de Souza & de Chaves, 2007; Vazzoler, 1996). Six maturation stages were defined for both females and males:

- Immature (A): females − filiform, translucent ovaries, very small in size, without signs of vascularization; macroscopically, oocytes are also not observed; males − small and filiform testicles.
- Early maturation (B1): females − ovaries with increased size and circumference, slightly yellowish colour, poorly vascularized and the presence of small oocytes; males − testicles increased in size compared to the previous stage, flatter compared to ovaries and whitish.
- Final maturation (B2): females − ovaries much larger than the previous stage, with dark yellowish colour, very close in size to mature ovaries, but with the presence of opaque and small oocytes; males − well‐developed, lobulated testicles, whiter in appearance, with a membrane that ruptures with slight pressure.
- Mature (C): females − size slightly larger than the previous stage, occupying almost the entire coelomic cavity; oocytes are yellowish, with a haemorrhagic appearance, and quite cylindrical; males − appear turgid, whitish, occupying a significant portion of the coelomic cavity, reaching their maximum size, with a milky white colouration.
- Empty (D): both decrease in size; they exhibit varying degrees of flaccidity, with stretched membranes, and few oocytes are observed in the case of females.
- At rest (E): both occupy less than one‐third of the coelomic cavity, reverting to the characteristics of the immature or early maturation stages.

Juveniles, if encountered, were classified when the gonads could not be unequivocally assigned to either the male or female category (Vazzoler, 1996).

### 2.3 Data analysis

The maturation stages were macroscopically evaluated using a stereomicroscope, and the gonadosomatic index (GSI), DI and modified gonadosomatic index (MGSI) were estimated to assess which one is more appropriate for the sampled population of *C. gariepinus* through Spearmen correlation with TL. TL is used as a reference for gonadal maturation (Binohlan & Froese, 2009). The GSI was calculated using the formula GW × 100/EW, where GW is the gonad weight and EW is the eviscerated fish weight (Vazzoler, 1996). The MGSI was calculated using the formula MGSI = 100 × GW/BW − GW (Nikolsky, 1963) and the DI was calculated using the formula DI =^3^√GW (Dobriyal et al., 1999), where BW is the fish weight, and GW is the gonad weight of the fish. We also calculated the hepatosomatic index (HSI) using the formula LW/EW × 100, where LW is the liver weight and EW is the eviscerated fish weight (Vazzoler, 1996). Fulton's condition factor is referred to as the ‘K factor’, assuming the isometric growth of the fish calculated as K=BW/TL^3^, where K is Fulton's condition factor; 3 is the exponent indicating isometric growth, TL is the total length, BW is the total fish biomass (Cren, 1951).

Due to the small sample size, robust statistical methods were used to identify patterns with higher confidence. Descriptive analyses were complemented by nonparametric tests (Mann–Whitney U test, Kruskal–Wallis test and Spearman's correlation), which are suitable for non‐normal distributions and limited data. Spearman's correlation was also employed to explore relationships between the HSI, the most sensitive reproductive indicator for this invasive population, and the condition factor. According to Htun‐Han (1978), these three indices can help assess the fish's nutritional status and its influence on the reproductive cycle. Mann–Whitney U tests were applied to compare differences in indices between sexes and across sampling periods. Complementarily, correlations were performed between the observed maturation stages and the three calculated reproductive indices (GSI, MGSI and DI) to assess whether these indices are good indicators of gonadal maturation.

The measured abiotic variables were summarized using principal component analysis (PCA), which was applied to the environmental data matrix at the catfish capture sites to assess temporal distribution patterns of the population and to reduce the dimensionality of the abiotic variables before correlating them with reproductive indices. In addition, a permutational multivariate analysis of variance (PERMANOVA) was applied to test whether differences in the distribution pattern of the catfish (presence/absence), as well as in its abundance (N), in relation to abiotic variables and vegetation cover characteristics, differed between the dry and rainy periods. Bray–Curtis distance was used in all PERMANOVA tests, with 4999 permutations, as recommended by Kemp and Manly (1997).

Due to the low number of captures of the invasive species, precise correlations between the measured abiotic water variables and the GSI could not be detected using traditional regression models. Consequently, the abiotic variables were modelled against the most sensitive reproductive index for this population using boosted regression trees (BRT), thereby identifying which factors may influence the reproduction of *C. gariepinus*. According to the procedure outlined by Elith et al. (2008), the BRT model was fitted with a tree complexity of 10, a learning rate of 0.001, a bag fraction of 0.75 and a Gaussian error distribution [for more details on these parameters, refer to Elith et al. (2008)]. Ten‐fold cross‐validation was applied to address the non‐independent structure of the data (Buston & Elith, 2011; Fabricius & De'ath, 2008). All data analyses were conducted in the R environment (R Core Team, 2025).

## 3 RESULTS

**FIGURE 2:**
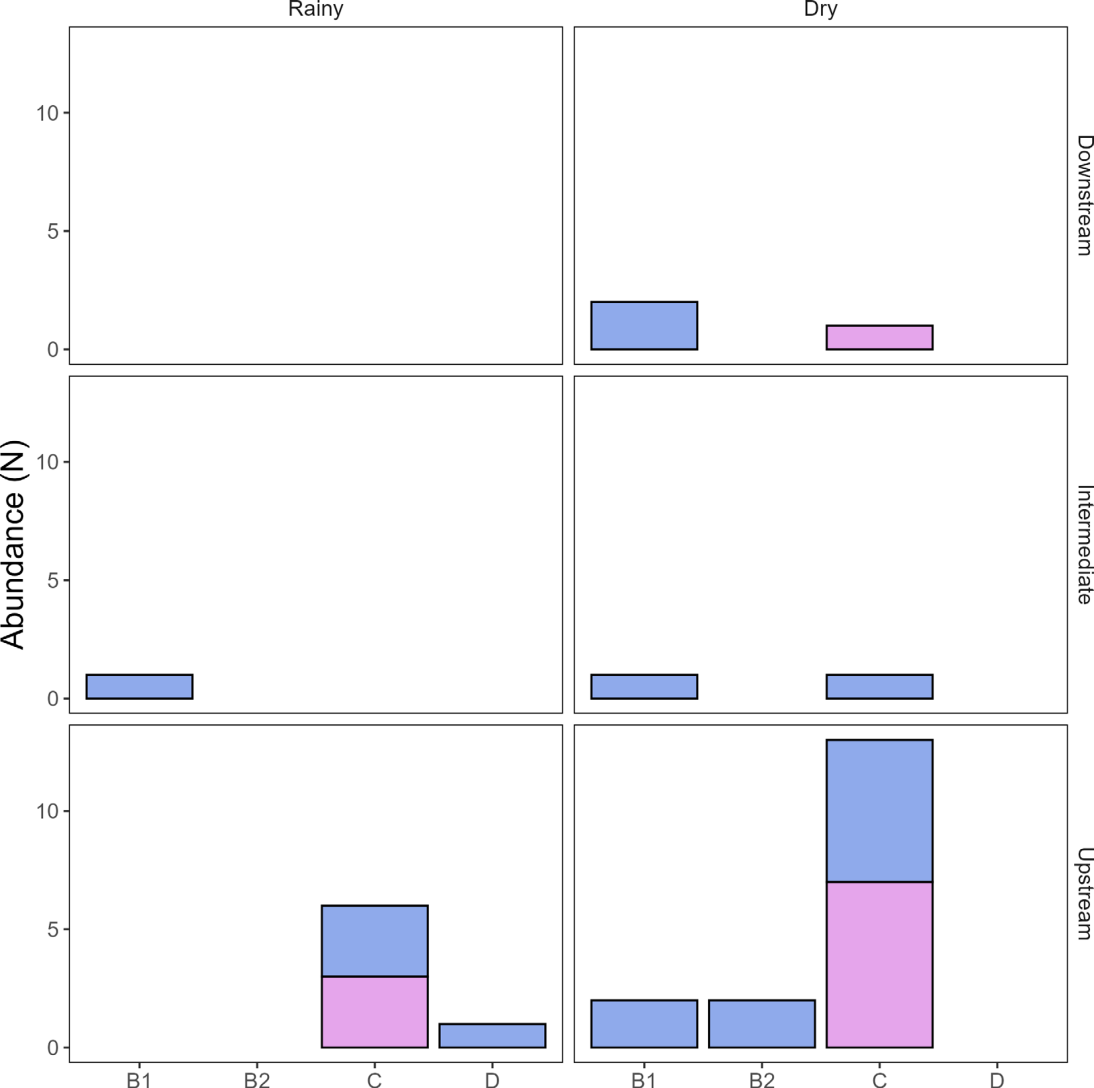
Maturation stages of *Clarias gariepinus* specimens during the rainy and dry seasons in the three stretches (downstream, intermediate and upstream) of the Guapi‐Macacu River.

**FIGURE 3:**
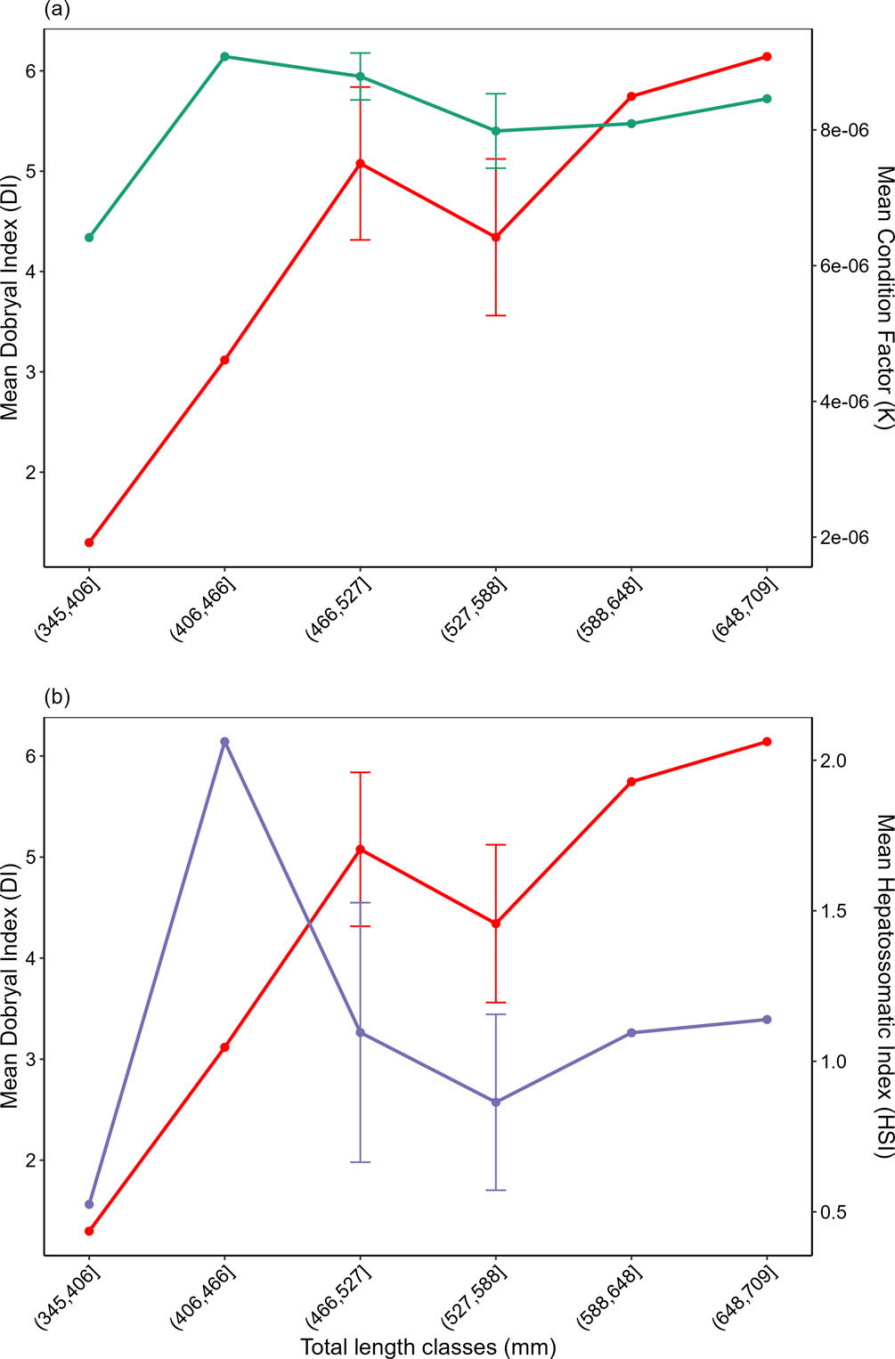
Variation in the condition factor (green line, panel a) and the hepatosomatic index (blue line, panel b) in relation to the Dobriyal Index (red line) for female individuals of the *Clarias gariepinus* population across size classes (closed interval: parentheses, open interval: square brackets).

**FIGURE 4:**
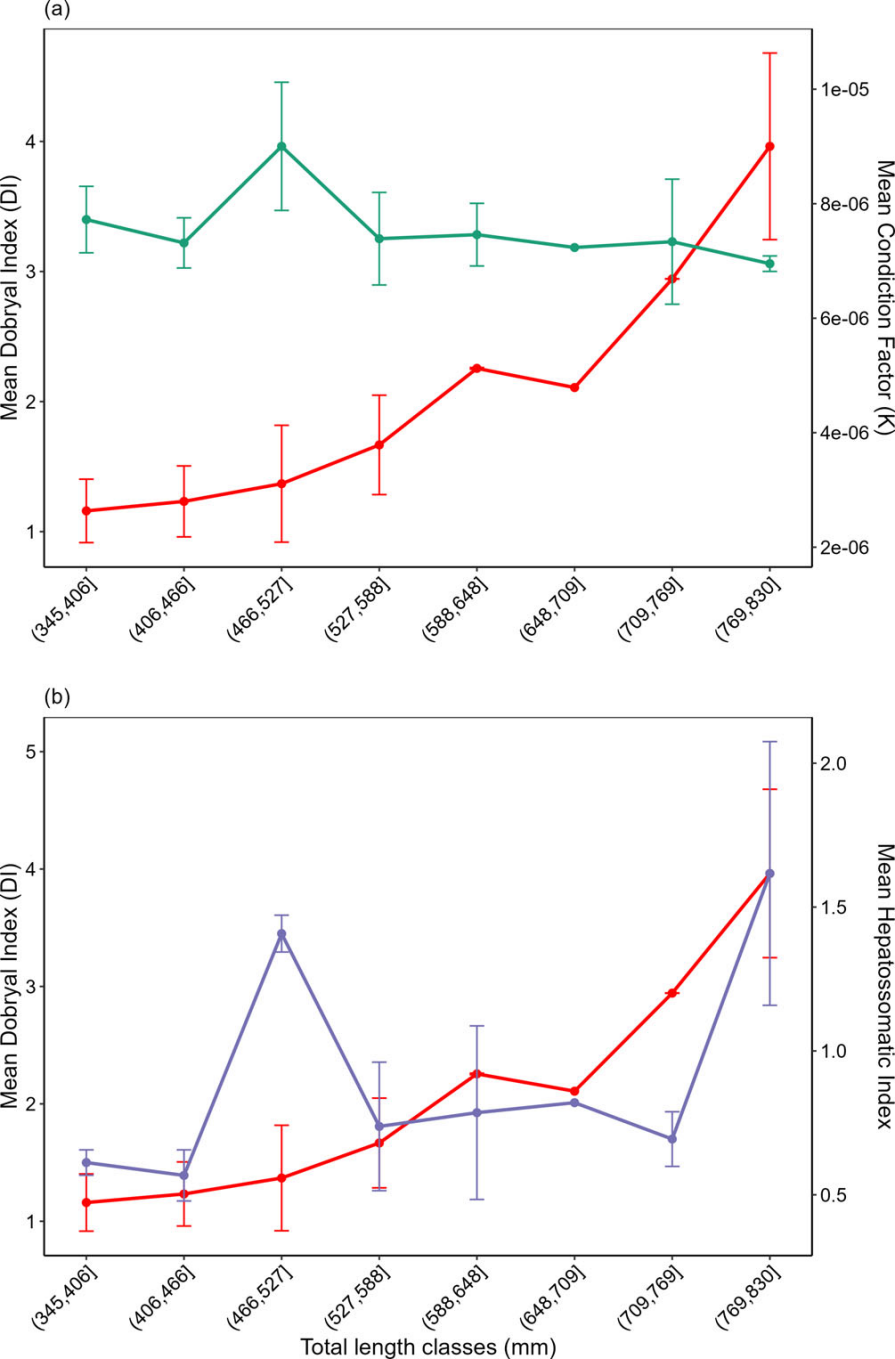
Variation in the condition factor (green line, panel a) and the hepatosomatic index (blue line, panel b) in relation to the Dobriyal Index (red line) for male individuals of the *Clarias gariepinus* population across size classes (closed intervals: parentheses; open intervals: brackets).

Thirty specimens of *C. gariepinus* were captured, predominantly males (63.3% M and 36.7% F), all adults at different stages of maturation (Figure 2), mainly upstream of the Guapi‐Macacu River; the dry season contributed to the majority of the collections (73.3% during the dry period and 26.7% during the rainy season), as shown in Table 1. For females, the weights ranged from 366 to 2432 g, with a TL ranging from 385 to 660 mm (Supporting Information S1). For males, the weight ranged from 334 to 4030 g, with the TL of 345–830 mm (Supporting Information S1). The condition factor showed higher means for females in larger size classes, except for lengths between 527 and 588 mm, whereas the DI was higher in the larger size classes (Figure 3a). The HSI exhibited an inverse relationship with DI, with higher averages in smaller size classes (Figure 3b). For males, smaller individuals showed a higher condition factor compared to DI (Figure 4a). However, males showed a single peak in the HSI between the size classes of 466 and 588 mm, with low values in all other samples (Figure 4b).

**TABLE 1:** Gonadal maturation stages of male (M) and female (F) specimens collected during the dry and rainy seasons in the upstream (U), intermediate (I) and downstream (D) sections of the Guapi‐Macacu River.

Zone	Sex	B1	B2	C	D
D	M	2	‐	‐	‐
	F	‐	‐	1	‐
I	M	2	‐	1	‐
U	M	2	2	9	1
	F	‐	‐	10	‐

All three indices showed no significant differences between the rainy and dry seasons (Wilcoxon rank‐sum test, *p* >0.7, for all), indicating that the captured specimens were in a reproductive period in both seasons. Similarly, when the three indices were compared among river stretches, no significant differences were observed (Wilcoxon rank‐sum test, *p* >0.7 for all), indicating the same reproductive pattern along the river. Among the gonad‐weight–based indices, only the DI exhibited a significant Spearman correlation with TL of the collected specimens, indicating that this index is more sensitive for this *C. gariepinus* population (Table 2). For the correlation between the calculated indices (GSI, MGSI and DI) and the maturation stages, none showed a significant correlation (Spearman, *p* >0.05, for all), with the greatest magnitude observed for the DI index (*Rho* >0.29).

**TABLE 2:** Spearman's rank correlation between reproductive indices GSI, DI, MGSI and the total length of *C. gariepinus* specimens in the Guapi‐Macacu River.

Index	*Rho*		*p‐Value*	
	Males	Females	Males	Females
GSI	0.5701	0.2727	0.001	0.4182
DI	0.8368	0.6545	<0.001	0.0383
MGSI	0.5877	0.3	0.009354	0.3711

Abbreviations: DI, Dobriyal Index; GSI, gonadosomatic index; MGSI, modified gonadosomatic index.

During the dry season, which recorded the highest number of samples, a significant correlation (*p* <0.05) was observed between DI and HSIs as well as a similar trend in correlation between the DI and the condition factor (Table 3). Conversely, during the rainy season, no significant results were obtained (*p* >0.05), and the explanatory capacity of these correlations was limited. Overall, there appears to be a more notable correlation between DI and the calculated condition factor during advanced stages of gonadal development.

**TABLE 3:** Spearman's rank correlation between the Dobriyal Index (DI) and the hepatosomatic index (HSI) and condition factor (K) of collected *C. gariepinus* specimens during dry and rainy seasons in the Guapi‐Macacu River.

	*Rho*		*p‐Value*	
	Dry season	Rainy season	Dry season	Rainy season
HSI	0.5787	0.4285	0.0055	0.2992
K	0.5019	−0.0476	0.0185	0.9349

**FIGURE 5:**
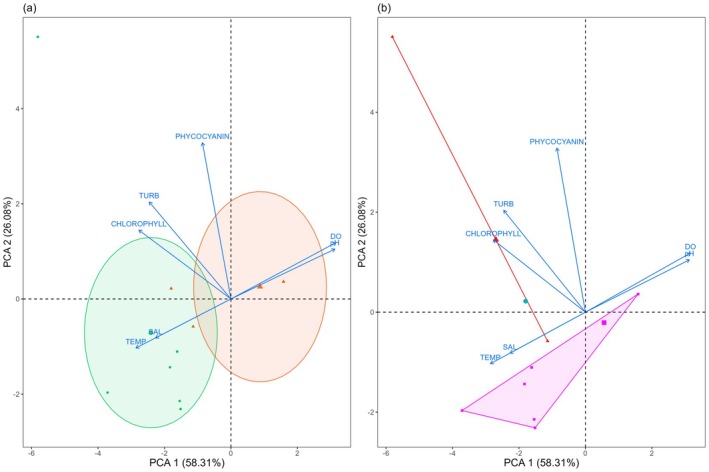
Principal component analysis (PCA) showing the percentage of inertia explained by the first two axes and the explanatory variables for the distribution of *Clarias gariepinus* by period (a) and by river sections (b) in the Guapi‐Macacu River. Panel A: dry period (orange); rainy period (green). Panel B: upstream (pink); intermediate (red); downstream (green).

The characteristics of the abiotic factors measured in the Guapi‐Macacu River indicate that the African catfish population shows stronger associations with pH, dissolved oxygen and temperature, which are shaped by the dry and rainy periods and by the river's physiographic features (Table 4). This pattern is supported by significant differences in abiotic variables and vegetation cover characteristics between the dry and rainy periods, both in relation to catfish presence and abundance (F = 11.719; *p* <0.001 for both). In the abiotic variables measured at the collection points along the river, higher averages for temperature were evident during the rainy season, associated with the warmer period in southeastern Brazil (Supporting Information S2a). pH behaved oppositely to temperature, favouring more acidic waters during the warmer and rainy periods (Supporting Information S2b). Turbidity exhibited higher averages during the rainy season, although the upstream stretch showed little variability between periods (Supplement S2c). The rainy season was responsible for displaying the lowest averages of dissolved oxygen in the intermediate and downstream sections of the river (Supplement S2d). Low salinity was characteristic in all three collection sections, except during the dry period downstream, where salinity intrusion was recorded in this section of the river (Supplement S2e). Phycocyanin and chlorophyll can infer the quality of the Guapi‐Macacu River and be correlated with turbidity, reaching maximum values during the rainy season downstream (Supplement 2f,g). PCA highlighted the complex relationships between environmental variables in the distribution of the African catfish (Figure 5a,b), with the first axis having major contributions from pH (20.58%), dissolved oxygen (21%), temperature (17.19%) and chlorophyll (16.12%). On the second axis, the abiotic variables with major contributions were turbidity (19.52%) and phycocyanin (50.71%). The river's physiographic characteristics are presented in Table 4.

**TABLE 4:** Average percentage (%) of vegetation physiography within each of the spatial areas corresponding to the upstream (U), intermediate (I) and downstream (D) sections of the Guapi‐Macacu River.

Zone	Tree	Mangrove	Grass	No_Veg
D	35.00%	50.00%	‐	15.00%
I	23.33%	48.33%	18.33%	10.00%
U	0.08%	16.96%	59.00%	23.96%

**FIGURE 6:**
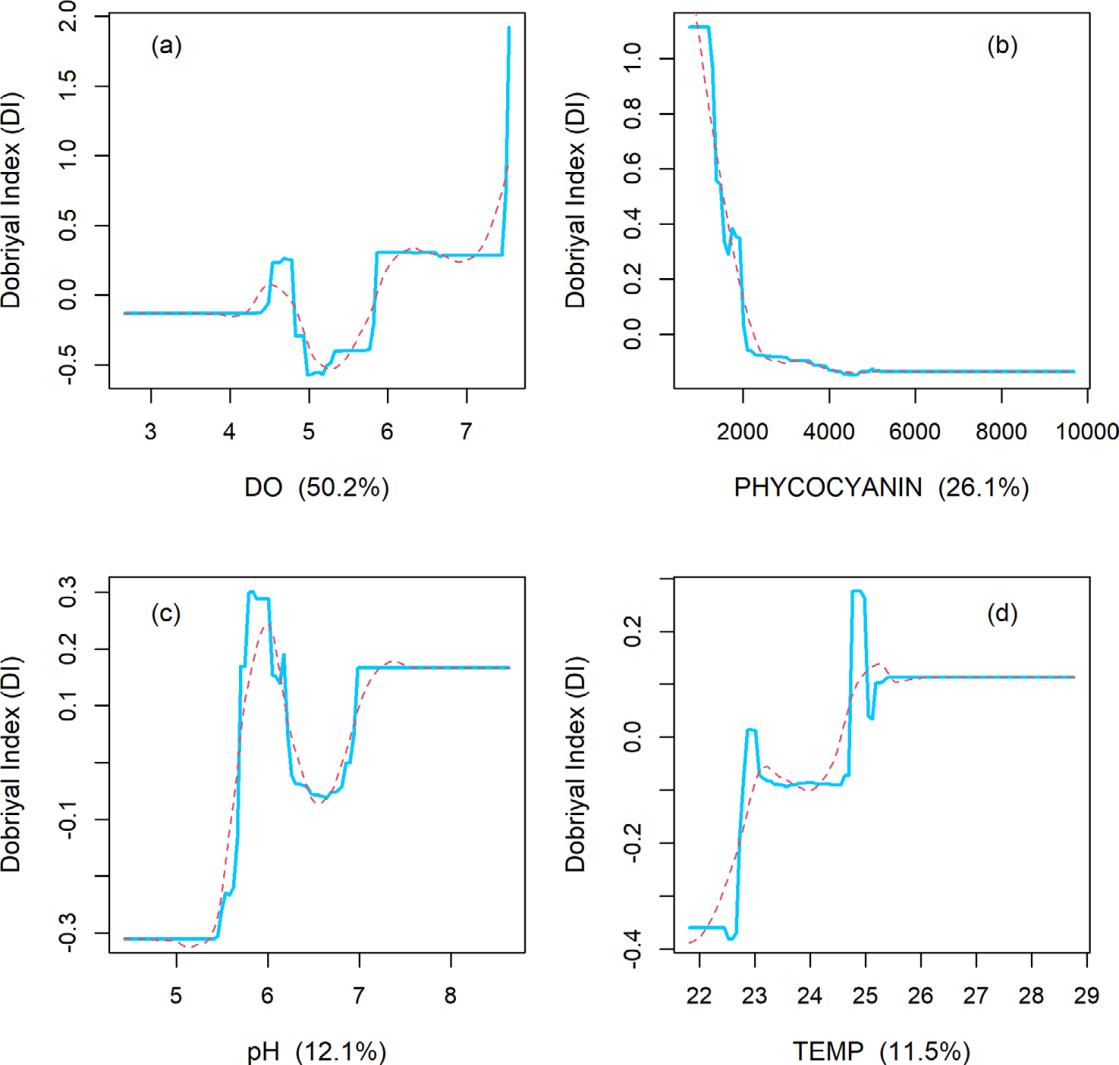
Relative importance showing the percentage contributions of the predictors dissolved oxygen (a), phycocyanin (b), pH (c) and temperature (d), in predicting variations in the Dobriyal Index for *Clarias gariepinus* using the boosted regression trees (BRT) model. Fitted functions for each term in the model are shown with BRT main effects ordered by relative influence. Solid blue lines represent the raw model‐predicted response in relation to the *X*‐axis, whereas dashed red lines indicate model smoothing (*Loess* curve) based on predictions made from 1000 models fitted to bootstrap samples of the fish data.

The BRT model demonstrated an overall satisfactory fit to the data, as evidenced by the absence of overfitting, indicated by the average total deviance (3.249) and average residual deviance (1.316). This suggests a capacity to explain approximately 59.9% (*R* ^2^) of the total variability in the DI. However, it is important to note that other factors may not be considered in the model contributing to the remaining variation. Cross‐validation (0.597; SE = 0.113) supported this adequate generalization to new datasets not used during training, explaining 57.4% (adjusted *R* ^2^) of the cross‐validated data. This indicates that the model can make reliable predictions. Figure 6 illustrates the model fit for the relative influence of six abiotic variables. Dissolved oxygen (50.2%) exhibited a positive relationship with the DI with higher values. Phycocyanin (26.1%) showed a decreasing influence on the DI. The pH (12.1%) was associated with an increase in the DI at pH values around 6 and at values above 7. Water temperatures of 23C and 25°C were associated with peaks in the DI, with a relative influence of 11.5% when considering the remaining set of variables.

## 4 DISCUSSION

In general, the reproductive population dynamics of fish are more accurately assessed when greater capture effort is applied over time (Singh, 2021). However, due to the constant difficulty in capturing the exotic species African catfish in various invaded areas (Erarto & Getahun, 2020), a more intensive sampling effort was chosen in the cleanest river of the region's watershed (Rangel et al., 2016). Thus, the 64 established collection points along the river allowed for the collection of 30 *C. gariepinus* samples in just two campaigns. However, Weyl et al. (2016) emphasize the scarcity of empirical studies on the reproductive cycle of African catfish in invaded habitats, a gap that the current study helps to address.

It was not possible to determine the length at first maturation of the species in this environment due to multiple factors, which may be correlated as discussed hereafter. According to Willoughby and Tweddle (1978), juveniles of *C. gariepinus* of approximately 1 year of age can be captured with fine‐mesh nets during January and February, although they are difficult to find in natural environments. However, this period coincides with the highest flood levels of the system, when increased river volume and turbidity promote greater flooding of the mangrove. This substantially increases the availability of refuge and shelter areas for juveniles while simultaneously limiting access for the sampling methods employed. This pattern was observed at the sampling stations, as the upstream area of the river provided most of the specimens, which were mostly caught using traps, where the mangrove is absent. Additionally, in Brazil, the sampling of juveniles in invaded ecosystems has been comparatively lower than that of adult specimens (Weyl et al., 2016), a pattern also reported for India (Tilahun et al., 2016).

Despite the absence of juveniles in the samples, most collected specimens were mature, in the C stage of gonadal maturation, indicating that the species finds a conducive environment for reproduction. In this sense, the absence of juveniles may be interpreted as a limitation of detectability rather than a lack of recruitment, which is consistent with the role of the mangrove as a refuge area within the river system. Moreover, the documented maximum age of 25 years for the species (Ellender et al., 2015; Wartenberg et al., 2013) suggests that the African catfish currently present in the protected area are part of a naturalized population with active reproduction. Wartenberg et al. (2013) emphasize that the presence of long‐lived fish (up to 25 years) may favour rapid establishment and long‐term persistence, thereby determining the success of the invasion. Accordingly, the occurrence of mature adult individuals, combined with the species' high longevity, suggests that the population present in the protected area is naturalized and persists over time, even in the absence of quantitative historical data that would allow the assessment of population trends, likely dating back to the 1980s.

*C. gariepinus* exhibits a more favourable reproductive period in its natural habitat during the rainy season and with an extended photoperiod (Bruton, 1979; Olaleye, 2005; Romanova, Lyubomirova, Romanov, Mukhitova, Shlenkina, Shadieva, & Galushko, 2018). On the contrary, the species is also observed with mature individuals throughout the year in various ecosystems (Legendre et al., 1996; Nwadukwe & Ayinla, 1993), consistent with our findings. The observed reproductive plasticity suggests an extended spawning period, or multiple peaks throughout the year (e.g., elevated fecundity during the rainy season). This flexibility aligns with the conceptual framework of reproductive plasticity in invasive fishes: substantial temporal variation in reproductive indices reflects the ability to adjust reproductive output in response to fluctuating environmental conditions possibly (García‐Berthou, 2007; Klarl et al., 2024).

Despite appearing numerically low, the capture of the African catfish is quite prominent compared to other Brazilian ecosystems (Weyl et al., 2016), where the alien species has significantly impacted the aquaculture community (Alves et al., 2007). Overall, a decline in fish fauna has been observed in the region in recent years. In 2006 (ICMBio), the Brazilian Ministry of the Environment recorded approximately 167 fish species in the Guapimirim Environmental Protection Area and Guanabara Bay (81 marine and 86 freshwater species). In contrast, in 2018, only 31 fish species were recorded, with a higher proportion of rare species (Dumith & Santos, 2022). In the same study, the IAS was notably abundant compared to native species. Migratory species were the most prevalent in the Guapi‐Macacu River, with the African catfish being the most abundant among freshwater species (Dumith & Santos, 2022). In addition to the poor water quality observed, the alien catfish may contribute to this decline, potentially impacting native fauna (Vitule et al., 2009; Weyl et al., 2016) while maintaining its population in the area since the 1990s.

The higher percentage of male African catfish may highlight the role of temperature as a determinant of sex differentiation (Santi et al., 2016; Santi et al., 2017; Santi et al., 2019). Although females are most commonly used for reproductive studies (Ataguba et al., 2012), *C. gariepinus* is a species that has been extensively studied due to its widespread use in aquaculture (Lisachov et al., 2023). Consequently, numerous studies have been conducted to characterize its reproductive biology (Al‐Deghayem et al., 2017; Arome Ataguba et al., 2013; Nazneen et al., 2021; Olaleye, 2005), facilitating reproductive characterization in both sexes (Adebayo et al., 2012; Arome Ataguba et al., 2013) and revealing distinct features between captive and wild populations (Müller et al., 2019; Olaleye, 2005). Thus, although males and females occur in different proportions in our study, reproductive rates tend to follow a similar pattern for both sexes, ensuring fecundity due to the reproductive synchrony of the sexes. Regarding gonadal maturation stages, the study conducted by Singh et al. (2021) observed greater variability in gonadal maturation stages for males than for females in natural environments, which confirms our findings. These authors also suggest that variation across different reproductive phases, as well as in the GSI and gonadal hormones, contributes to successful colonization and establishment in fluvial environments such as the Ganges River (India). On the contrary, there is evidence that the rate of gonadal maturation in this species varies with geographic location, in addition to environmental factors (Goos & Kichter, 1996). Thus, the lack of pronounced seasonal and spatial patterns in the calculated reproductive indices (GSI, MGSI and DI), coupled with the significant sex‐related differences, is consistent with opportunistic species exhibiting broad environmental tolerance. These findings support the hypothesis that *C. gariepinus* demonstrates continuous reproduction and reproductive plasticity, traits that confer ecological robustness and competitive advantage in this environment.

Among the calculated indices to assess the alien species, the DI showed the strongest relationship with fish length, likely due to its greater sensitivity, considering only gonad weight (Rayal et al., 2021). This contrasts with other indices that take into account the total weight of the fish. Indeed, there was a better correlation between DI and the condition factor for females, which can be explained by the fact that eggs constitute 15%–20% of the body weight (Megbowon & Fashina‐Bombata, 2013), due to advanced stages of gonadal development. The IGS is widely employed in various studies, often utilized for calculating the size at first maturation. However, discrepancies exist in its calculations. Conversely, the MGSI is infrequently employed, much like the DI, yet it appears to reflect similar patterns to the IGS. The study conducted by Rayal et al. (2021) compared the DI and the IGS of *Puntius ticto*, an ornamental fish found in the Aasan River in India. The study demonstrated that the DI was the most appropriate for the species, corroborating the findings of Esmaeili and Shiva (2006). In their study Esmaeili and Shiva (2006) compared the IGS, MGSI and DI to determine the gonadal first maturation length of *Aphanius persicus*.

In this manner, the DI was compared to other indices that infer the fish's energy reserves (glycogen) and ecological condition. It is observed that glycogen peaks precede the gonadal development phase. However, the condition factor tends to be lower during the peak of gonadal development. This may indicate that, despite a slightly lower ecological condition, the African catfish can store sufficient energy to sustain its gonadal development. The relationships between condition factors and HSI are well established (Nwadukwe & Ayinla, 1993). Our results indicate that fluctuations in ecological condition and HSI are more pronounced in smaller, presumably younger fish. Although overall condition declines with increasing TL, peaks in HIS (reflecting energy reserves) persist across size classes (Abdel‐Warith et al., 2014). These HSI peaks correspond to elevations in the DI, thereby sustaining continuous reproduction irrespective of body size or sex. Thus, the observed values of the reproductive indices (DI, GSI and MGSI) for *C. gariepinus* indicate continuous reproduction, irrespective of aquaculture provenance, in contrast to cultured populations where spontaneous spawning does not occur (Colautti et al., 2006; Romanova, Lyubomirova, Romanov, Mukhitova, & Shlenkina, 2018). Therefore, environmental conditions may play a determining role for the species, such as variations in river levels and other abiotic factors (Abdel‐Latif et al., 2021). Nevertheless, the species' resilience characteristics were also evident in these growth relationships, even with a low condition factor. In captive environments (aquaculture), it tends to maintain growth under conditions where abiotic factors are unfavourable (Damar et al., 2020), where many fishes could otherwise perish.

The alluvial plains within the Environmental Protection Area, which become inundated during the rainy season, tend to exhibit reduced dissolved oxygen levels owing to the dense mangrove canopy Although *C. gariepinus* is known to survive under low dissolved oxygen conditions owing to its accessory respiratory organ (Ip et al., 2004), our results indicate that the species' reproduction remains dependent on favourable physical parameters, particularly water oxygenation. Accordingly, its migratory behaviour in pursuit of optimal pH and dissolved oxygen levels is consistent with the patterns described by Mbalassa and Nshombo (2020), exemplifying the dynamic interplay between abiotic factors and reproductive processes. Comparable observations were reported by Klarl et al. (2024) for invasive gobiid fishes, whose reproductive output proved highly responsive to local climatic drivers, thereby highlighting plasticity as a pivotal trait facilitating reproductive expansion in novel habitats. In this context, our findings for *C. gariepinus* (i.e., evidenced by the uniformity of reproductive patterns along the environmental gradient) underscore the species' capacity to efficiently exploit the spatial heterogeneity of the riverine system. Moreover, the structural complexity of the mangrove habitat further impedes fish capture in both the dry and rainy periods.

Thus, when we correlate DI with abiotic variables, we observe that these differ somewhat from the relationships indicating the relative influence of these same factors. The most significant variations in pH, found to align with the greatest variations in dissolved oxygen during the dry season, were selected for the distribution of specimens. These variations were preferred in sections where better conditions were observed, particularly upstream (Pokharel et al., 2018), highlighting the higher percentage of specimens encountered (Pease et al., 2012). The pH is a crucial abiotic factor for fishes, the variation of which can influence the regulation of ammonia excretion (Wilkie & Wood, 1996). For the BRT model, despite its low relative influence, our findings suggest that values close to a pH of 6 may favour its reproductive peak, aligning with previous studies described for the species (Aigbogun et al., 2015; Marimuthu et al., 2019). However, dissolved oxygen exhibited a more pronounced relative influence on DI than pH. *C. gariepinus* is often found in conditions of deficient dissolved oxygen levels in the water (Ariole et al., 2021; Chris et al., 2022; Oké & Goosen, 2019), and even in mud at times (Papuc et al., 2019), owing to its specialized aerial respiratory organ (Monteiro et al., 2021). It is possible, therefore, that the African catfish may require improved concentrations of dissolved oxygen for optimal gonadal development and reproduction.

Temperature is a critical factor for the reproduction of *C. gariepinus* (Müller et al., 2019; Romanova et al., 2020; Ukwe & Abu, 2016); however, our BRT model exhibited a low relative influence of this factor (11.5%), likely due to the already elevated temperature in the Guapi‐Macacu River throughout the year. The characteristics of temperature variations and the low amplitude of salinity variation were more pronounced during the rainy season, favouring the invasion and dispersion of this alien species in the river (Gutierre et al., 2014). This is linked to the fact that the African catfish triggers its migratory and reproductive cycles with an increase in water temperature (Kadye & Booth, 2013). Moreover, the period of intense rainfall is also associated with an increase in temperature in southeastern Brazil. Thus, the combination of these factors not only promotes the migrations and reproduction of the species but may have influenced the relationship with temperature in the BRT model. Additionally, the effects of climate change, already more pronounced in smaller water bodies (Pachauri & Reisinger, 2007; Rahel & Olden, 2008) may have contributed to the maturation of individuals, favouring early sexual differentiation (Bruton, 1979; Romanova et al., 2020; Santi et al., 2016). In this context, the relationship between the seasonal period and the reproductive season of the species is well associated with temperatures above 26°C (Romanova, Lyubomirova, Romanov, Mukhitova, Shlenkina, Shadieva, & Galushko, 2018), indicating sexual differentiation above 28°C (Santi et al., 2017) mainly found in the upstream area of the Guapi‐Macacu River. This may have favoured the higher number of male individuals in our findings.

Indeed, among all rivers within the Environmental Protection Area, this one exhibits the most favourable environmental conditions, although its water quality often deteriorates during certain periods due to multiple factors. (Costa et al., 2018). In this regard, the BRT model indicates an inverse relationship in the relative influence of environmental conditions associated with these three abiotic factors (the phycocyanin, showing a less favourable tendency towards the DI of the African catfish). On the contrary, conditions with better dissolved oxygen, temperature and pH are more conducive to the optimal development of the species' gonads. Thus, parameters associated with poor water quality are likely to impair the fish reproductive performance, suggesting that the species may also be vulnerable to further environmental degradation of the river (Dumith & Santos, 2023).

The reproductive patterns identified for *C. gariepinus* in the Guapi‐Macacu River indicate a high degree of invasive success, demonstrating the overcoming of potential invasion barriers, as highlighted by Weyl et al. (2016). According to these authors, a thorough understanding of the consequences of invasion is essential for the development of effective strategies to mitigate impacts and contain further spread. In addition, the longevity of the invasive population represents an additional factor promoting the successful establishment of this species, due to low mortality rates in the non‐native environment (Wartenberg et al., 2013). Thus, the knowledge generated in this study, particularly regarding the reproductive plasticity of the African catfish, may support the development of management and control programmes (Roshni & Taxa, 2020; Singh, 2021).

## 5 CONCLUSION

The results suggest that *C. gariepinus* exhibits remarkable reproductive plasticity and high environmental tolerance, which favour its expansion and persistence in invaded tropical environments. The use of the DI proved effective in detecting reproductive maturation patterns, regardless of body size, being particularly useful in limited sampling scenarios. In this context, the continuous gonadal activity throughout the year, as well as the preference for habitats with high levels of dissolved oxygen and optimal pH, highlights the role of these abiotic factors in modulating reproductive success. Additionally, the species appear to benefit from the constant high temperatures recorded throughout the year, which tend to intensify due to climate change. Considering the ability of African catfish to exploit favourable physicochemical conditions and maintain reproduction regardless of seasonality, the species represents a persistent reproductive threat to native ichthyofaunas. These findings support the hypothesis that physiological and longevity traits, such as robustness and environmental plasticity, are determinants of invasive success. These results also reinforce that successful reproduction in invaded environments is one of the main characteristics that distinguish successful IAS. Therefore, we recommend that monitoring programmes incorporate critical environmental indicators (dissolved oxygen, pH and temperature) in addition to management strategies that prioritize selective removal in locations identified as reproductive ‘hotspots’, minimizing the impacts of the invasion. Continuous investigations at multiple sites, focused on the life‐history traits of *C. gariepinus*, may further elucidate the mechanisms of its reproductive resilience and guide effective freshwater biodiversity conservation practices.

## AUTHOR CONTRIBUTIONS

**Michelle Torres Dumith:** laboratory analysis, data analysis and manuscript preparation. **Alejandra Filippo Gonzalez Neves dos Santos:** project conception, contributions to data collection, data analysis and manuscript preparation, supervision of the study.

## FUNDING INFORMATION

This study was performed within the scope of the project entitled ‘The Invasion of African Catfish in the Guanabara Bay – RJ’, contemplated by the National Council for Scientific and Technological Development (CNPq), Brazil, Universal Public Notice, no. 405984/2016–2. The author Michelle Torres Dumith received support from the Coordination for the Improvement of Higher Education Personnel (CAPES), no. 88887.644415/2021‐00.

## CONFLICT OF INTEREST STATEMENT

The authors declare that they have no conflict of interest. The present manuscript was not published or simultaneously submitted for publication elsewhere.

## Supporting information


**Supporting Information S1:** Box plot of total weight of *Clarias gariepinus* specimens during the rainy and dry seasons, across different stretches of the Guapi‐Macacu River. Blue: males; pink: females.

**Supporting Information S2:** Mean values (central square) with standard deviation for temperature (a), pH (b), turbidity (c), dissolved oxygen (d), salinity (e), phycocyanin (f) and chlorophyll (g) during the dry and rainy periods along the river. Y‐axis labels represent combinations of river sections (U: upstream; I: intermediate; D: downstream) and the dry (D) and rainy (R) periods.

## Data Availability

The data that support the findings of this study are available from the corresponding author upon reasonable request.
