# Supplementary figures and images for: Reproductive plasticity and environmental tolerance of invasive African catfish (Clarias gariepinus) in a tropical Brazilian river
Source: J Fish Biol. 2026 Mar 19;109(1):264–77. doi: 10.1111/jfb.70389 (PMC13397235; doi:10.1111/jfb.70389)

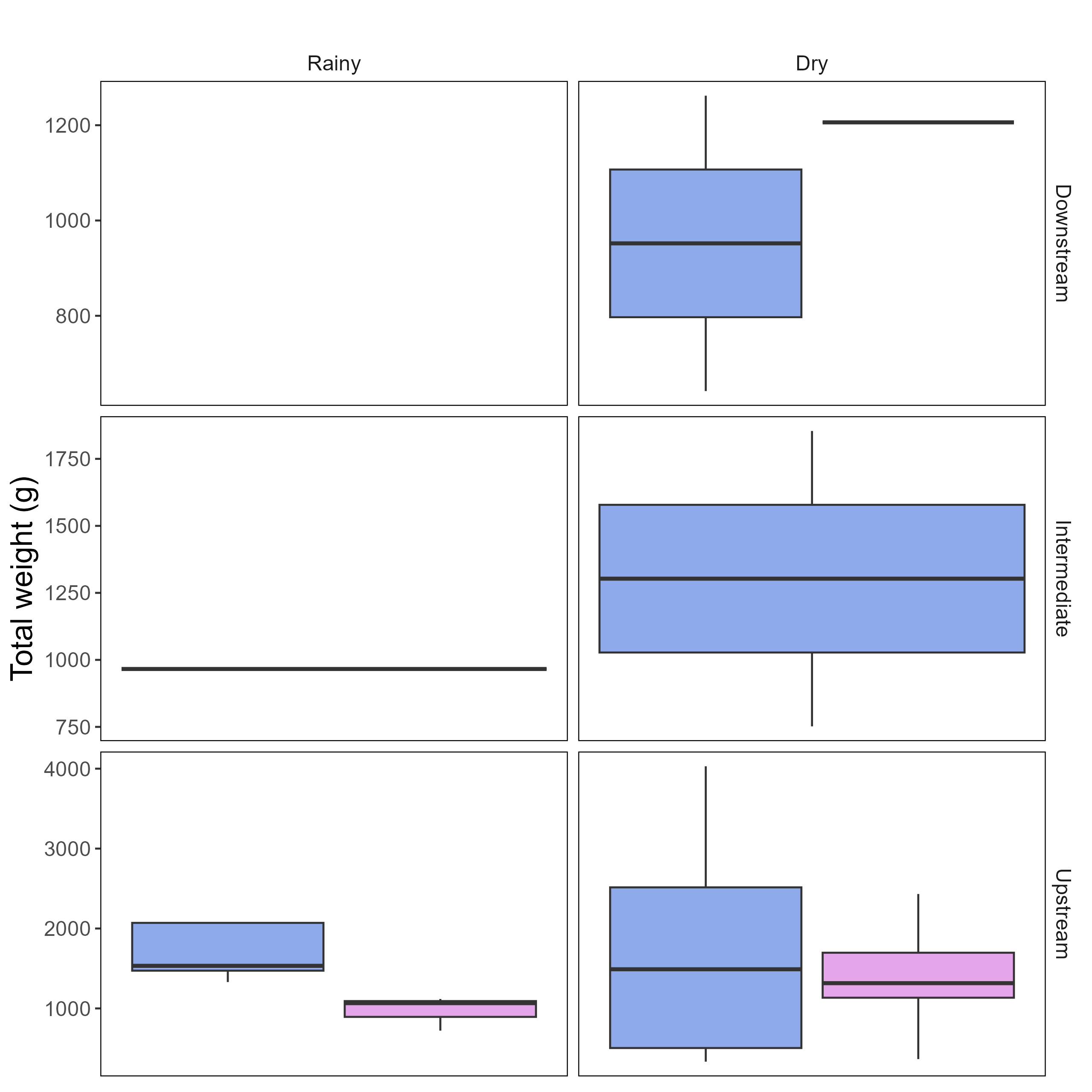

Supplement: Supplementary file 1 — Supporting Information S1: Box plot of total weight of Clarias gariepinus specimens during the rainy and dry seasons, across different stretches of the Guapi‐Macacu River. Blue: males; pink: females. [file JFB-109-264-s002.tiff]

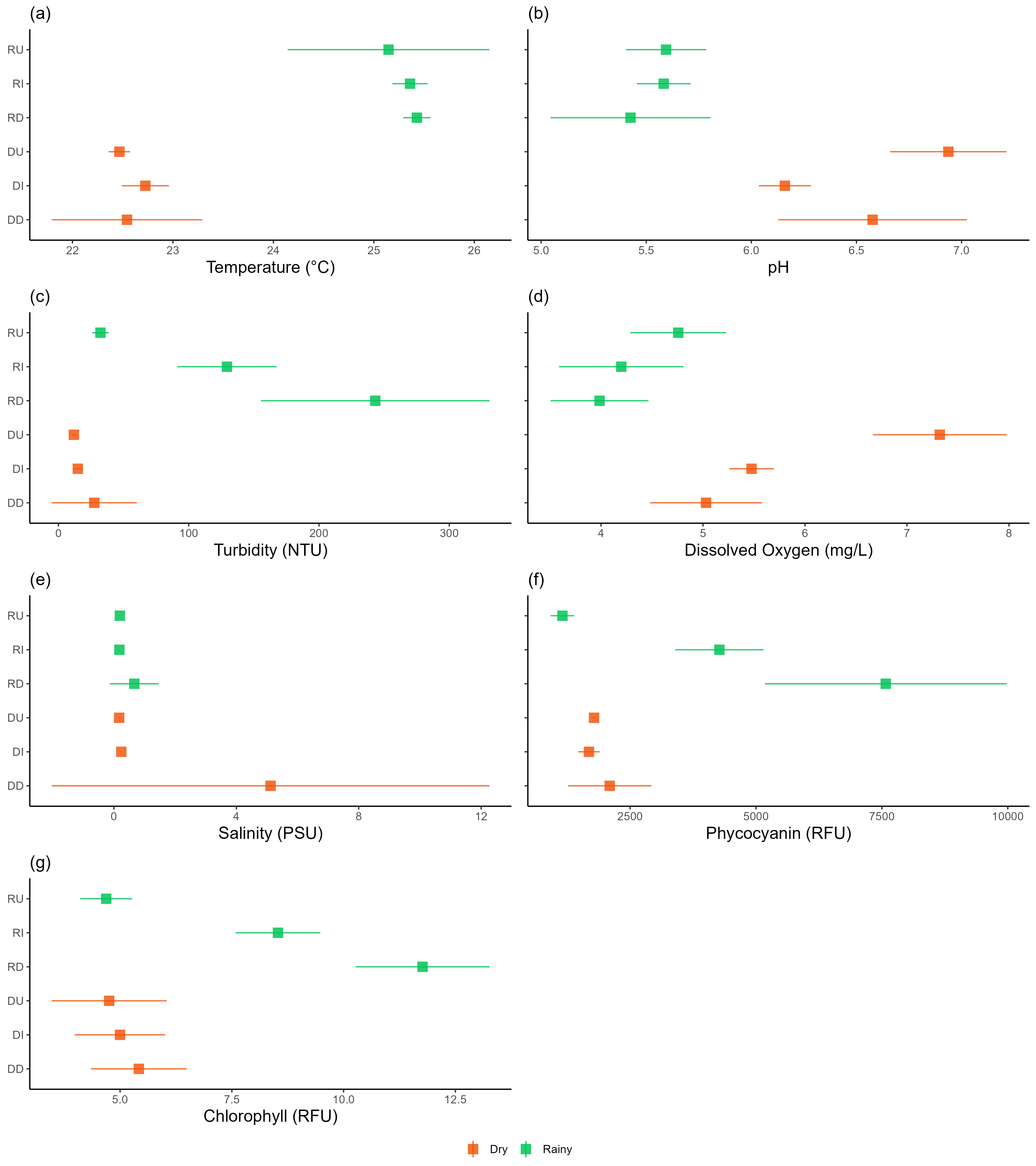

Supplement: Supplementary file 2 — Supporting Information S2: Mean values (central square) with standard deviation for temperature (a), pH (b), turbidity (c), dissolved oxygen (d), salinity (e), phycocyanin (f) and chlorophyll (g) during the dry and rainy periods along the river. Y‐axis labels represent combinations of river sections (U: upstream; I: intermediate; D: downstream) and the dry (D) and rainy (R) periods. [file JFB-109-264-s001.tiff]
